# Vibronically Coherent Exciton Trapping in Monolayer WS_2_

**DOI:** 10.1021/acsnano.5c08533

**Published:** 2025-07-21

**Authors:** Yorrick Boeije, Anh Tuan Hoang, Juhwan Lim, Samuel D. Stranks, Manish Chhowalla, Eric Pop, Andrew J. Mannix, Akshay Rao

**Affiliations:** † Department of Chemical Engineering and Biotechnology, 2152 University of Cambridge, Cambridge CB3 0AS, U.K.; ‡ Department of Physics, Cavendish Laboratory, University of Cambridge; Cambridge CB3 0HE, U.K.; § Department of Materials Science & Engineering, 6429 Stanford University, Stanford, California 94305, United States; ∥ Department of Materials Science & Metallurgy, University of Cambridge; Cambridge CB3 0FS, U.K.; ⊥ Department of Electrical Engineering, Stanford University, Stanford, California 94305, United States; # Department of Applied Physics, Stanford University, Stanford, California 94305, United States

**Keywords:** transition metal dichalcogenide defects, exciton trapping, conical intersections, vibronic coherence, exciton−phonon coupling, defect engineering, defect photophysics

## Abstract

Defect engineering in transition metal dichalcogenide (TMD) monolayers enables applications in single-photon emission, sensing, and photocatalysis. These functionalities critically depend on defect type, density, spatial distribution, relative energy, and the dynamics of exciton trapping at the defect sites. The latter are mediated by coupling to optical phonons through mechanisms not yet fully understood. Traditionally, exciton or carrier trapping at defects in inorganic crystals has been described by incoherent multiphonon emission within the Born–Oppenheimer approximation—an approach that underpins the widely used Shockley–Read–Hall framework for nonradiative recombination. Here, we use impulsive vibrational spectroscopy to investigate exciton trapping in defect-modified monolayers of WS_2_ grown through metal–organic chemical vapor deposition. We find that the phonon coherences of the Raman-active A’ and E’ modes persist throughout the ultrafast (∼100 fs) exciton trapping process, indicating a continuous evolution of the excitonic wave function. This observation is consistent with a conical intersection-mediated trapping process, in which a potential energy surface crossing between the free and trapped excitonic states acts as a funnel to drive this nonadiabatic transition. Such a molecular-like, vibronically coherent mechanism lies beyond the Born–Oppenheimer approximation, in stark contrast to classical, incoherent trapping models in solids. Moreover, the faster dephasing of the E’ mode in the trapped exciton state compared to the free exciton suggests it acts as a vibrational coordinate that promotes the trapping process. These findings provide mechanistic insights into exciton–phonon interactions at defects in TMD monolayers and inform strategies for engineering quantum and energy functionalities.

## Graphical abstract

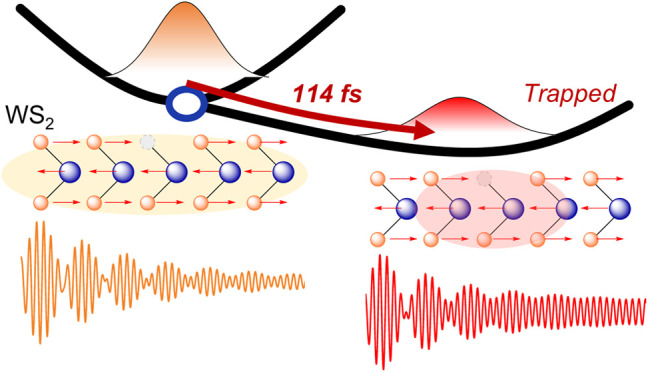

Defects are imperfections in crystal lattices that have historically been regarded as unwanted, hampering optoelectronic device performances in photovoltaics and light-emitting diodes by facilitating nonradiative recombination and reducing charge carrier mobilities. However, their inherently localized nature could be used to drive energy transfer^1^ and photochemistry^2^ for catalysis-based energy technologies or for single photon emission^3^ in quantum technologies. Two-dimensional (2D) transition metal dichalcogenides (TMDs) have emerged as a class of materials that could take advantage of such defect functionalities.^4^ The performance of defect-engineered TMD monolayers hinges on a deeper understanding of defect-specific exciton–phonon coupling, which is the main decoherence channel in single photon emitters,^5–7^ as well as the exciton trapping and recombination dynamics at those defects. Just as multiphonon emission models are used to describe free carrier trapping^8–12^ and nonradiative recombination (i.e., Shockley-Read-Hall processes)^13–71^ at deep defects in bulk semiconductors, similar theoretical frameworks have been developed to describe exciton trapping in TMDs.^16,17^

Experimental reports typically focus on exciton trapping and trap-related scattering mechanisms in TMDs but do not provide phonon mode-specific detail.^18–22^ Mode-specific detail is essential to trace exciton trapping pathways, yet such detail has remained experimentally inaccessible. While Raman studies offer phonon mode detail and, in some cases, can reveal local defect-induced modes,^23^ they are limited to ground-state structural characterization. Time-resolved pump–probe approaches offer the capability to study interaction of defects with phonons in the time domain, demonstrating a clear link between defect density and phonon dephasing^24,25^ and mode-selective interactions with defects.^26,27^ However, these experiments typically do not probe the full excited state absorption spectrum.

In this study, we employ broadband impulsive vibrational spectroscopy (IVS) to elucidate the mechanism of exciton trapping in TMD monolayers. IVS uniquely enables broadband probing of excited state vibrational modes^28,29^ and has proven powerful to identify key modes in ultrafast photochemical reactions,^30^ nonradiative decay in organic light emitting diodes^31^ and polaron formation in hybrid perovskites,^32^ yet it has not been applied to shine light on exciton/carrier trapping mechanisms.

Through a controlled, short-duration *n*-butyl-lithium (*n*-BuLi) treatment on WS_2_ monolayers grown by metal–organic chemical vapor deposition (MOCVD), we engineer a defect-rich model system tailored for a comprehensive study of exciton trapping at defect sites. The phonon coherences of the Raman-active A’ and E’ modes survive the ultrafast (∼100 fs) trapping process, indicating a continuous evolution of the exciton wave function. We demonstrate that vibronically coherent trapping is consistent with a conical intersection-mediated mechanism, where a crossing between potential energy surfaces associated with the free and trapped excitonic states acts as a funnel to drive the nonadiabatic transition. This interpretation is supported by the energetically distinct and bright defect emission, as well as the faster dephasing of E’ in trapped vs free excitonic population. These findings not only enhance our fundamental understanding of exciton–phonon interactions at defects but also provide critical insights for defect engineering strategies.

## Results

The preparation of untreated and *n*-BuLi treated MOCVD^33^ WS_2_ monolayer is described in the Methods section and schematically illustrated in Figure 1a. In addition to the well-known exciton A emission at 2.03 eV (*i* in Figure 1b), the *n*-BuLi treatment introduces a lower-energy emission (*ii* 1.88 eV) in the steady-state photoluminescence spectrum under ambient conditions (Figure 1b). In previous reports, the appearance of such a lower-energy bright emission after both annealing and chemical treatments have been assigned to defect excitons localized at sulfur vacancies,^34–36^ which introduce deep acceptor levels.^35,37,38^ Even though due to thermodynamic considerations it is expected that most sulfur vacancies will be filled with oxygen upon air exposure,^39^ it has been shown for MoS_2_ films with postgrowth treatment that sulfur vacancies can still persist at sufficiently high densities (>1 × 10^14^ cm^–2^).^34,40^ Importantly, oxygen substituted defects typically result in a blueshift of the exciton emission,^41^ as their levels align with the WS_2_ valence band, rather than the ∼0.2 eV red-shifted emission peak in this work and MoS_2_.^34^ Given the smaller energy separation relative to the *i* and *ii* emission peaks observed in the treated film, we assign the shoulder peak (1.95 eV) in the pristine film spectrum to trion emission, rather than defect-related emission, as previously reported for pristine films.^42^ Consistent with a reduction in trion population due to a suppressed n-type doping,^35,41^ the exciton A emission sharpens and exhibits a ∼30 meV blueshift following *n*-BuLi treatment. Based on the unchanged positions of the A’ and E’ Raman modes at 419 cm^–1^ and 355 cm^–1^ (Figure 1c), respectively, which match literature-reported WS_2_-monolayer spectra of the 2H phase,^43,44^ we can exclude any treatment induced phase change. The similar PL and Raman spectra of a *n*-BuLi treated mechanically exfoliated WS_2_ film illustrates the general applicability of this defect engineering method (Figure S1).

**Figure 1:**
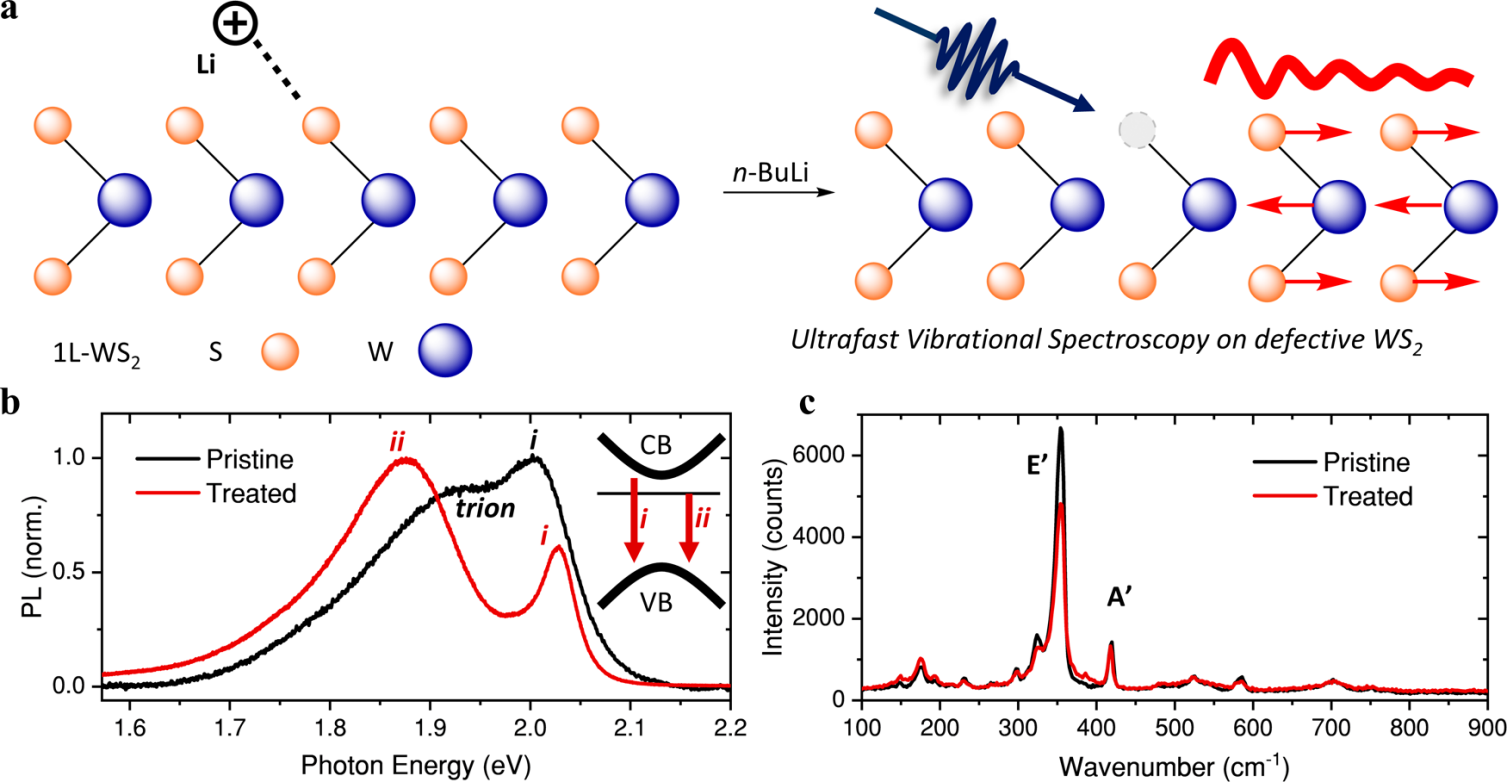
*n*-BuLi treatment of monolayer WS_2_induces a bright lower-energy emission. (a) Schematic of *n*-BuLi treatment. The proposed mechanism involves a positively charged lithium ion extracting a sulfur atom, leaving a sulfur vacancy. The interaction of vibrations with the defects are studied with ultrafast impulsive vibrational spectroscopy (IVS) in this work. (b) Photoluminescence spectra of pristine and treated WS_2_. The assigned emission peaks of the treated film are indicated in the inset. CB: conduction band. VB: valence band. (c) Steady-state Raman spectra of pristine and treated WS_2_. Optical measurements are taken at room temperature.

The transient absorption (TA) spectra of a pristine and a treated WS_2_ film are shown in Figure 2a,2b. The 600–625 nm spectral region is dominated by the exciton A bleach, and the 510–530 nm by the exciton B bleach, consistent with previously reported TA studies on monolayer WS_2_.^45–47^ To emphasize spectral differences between treated and pristine WS_2_ their differences (treated – pristine) are plotted in Figure 2c,2d, at 75 and 700 fs, respectively. The treatment induces a blueshift in the exciton A bleach, in agreement with the blueshift observed in the A exciton emission in Figure 1b, while the exciton B bleach remains unaffected. We took this blueshift into account by aligning the exciton A bleach of treated to pristine prior to subtraction, enabling a clearer comparison of relative bleach intensities. Importantly, right after photoexcitation (75 fs) the treated and pristine spectra are nearly identical, while the 700 fs difference spectrum shows a reduced exciton A bleach and an enhanced lower-energy bleach (670–720 nm) associated with the *n*-BuLi-treatment, consistent with ultrafast exciton trapping. A similar sub-ps growth of a subgap bleach has been observed by Bretscher et al.^35^ in sulfur vacancy containing MoS_2_. The broad 670–720 nm bleach agrees with the appearance of lower-energy emission feature (Figure 1b) corresponding to defect transitions.

**Figure 2:**
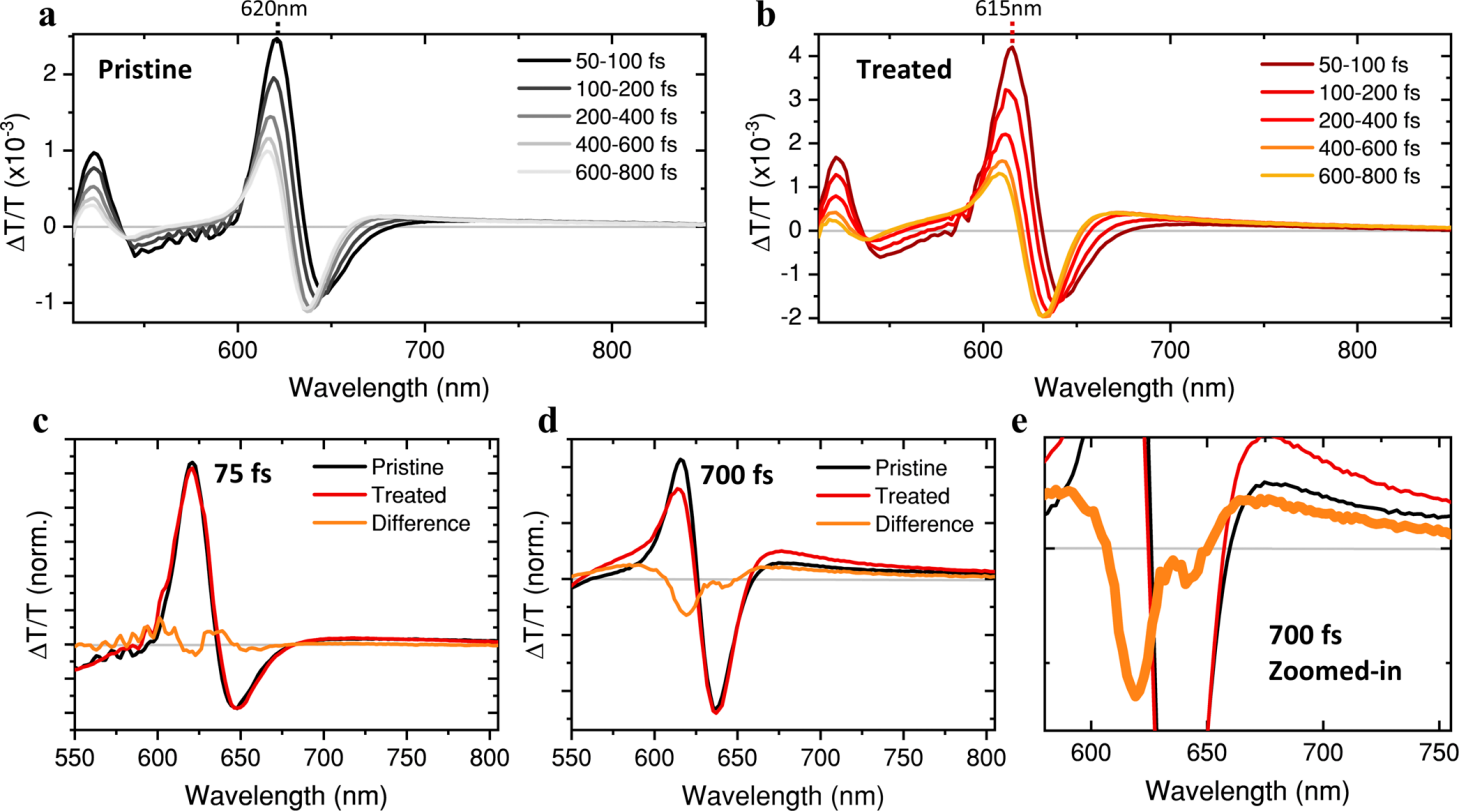
Ultrafast exciton trapping. (a) Femtosecond temporal evolution of the transient absorption spectra for a pristine WS_2_ film. (b) Transient absorption spectra for a *n*-BuLi treated WS_2_ film. (c) Difference spectra (after normalization of the full TA map) for both films at 75 fs. (d) Difference spectra at 700 fs. (e) Zoomed-in view of (d). The spectra in (c) and (d) are aligned to the exciton A bleach of the pristine film to account for the treatment-induced 5 nm blueshift. From the difference spectra the treatment induces a faster exciton A decay (600–625 nm), as well as a stronger lower-energy bleach (670–720 nm).

We focused our analysis on the spectral region corresponding to the A exciton bleach—hereafter referred to as the free exciton (Figure 3a)—rather than the exciton B bleach, due to lower signal-to-noise ratio of the latter. Although the trapped exciton bleach likely extends to wavelengths shorter than 670 nm, similar to what is observed in the defect emission, we limit our analysis to the 670–720 nm spectral region, where the TA signal is dominated by the trapped exciton.

**Figure 3:**
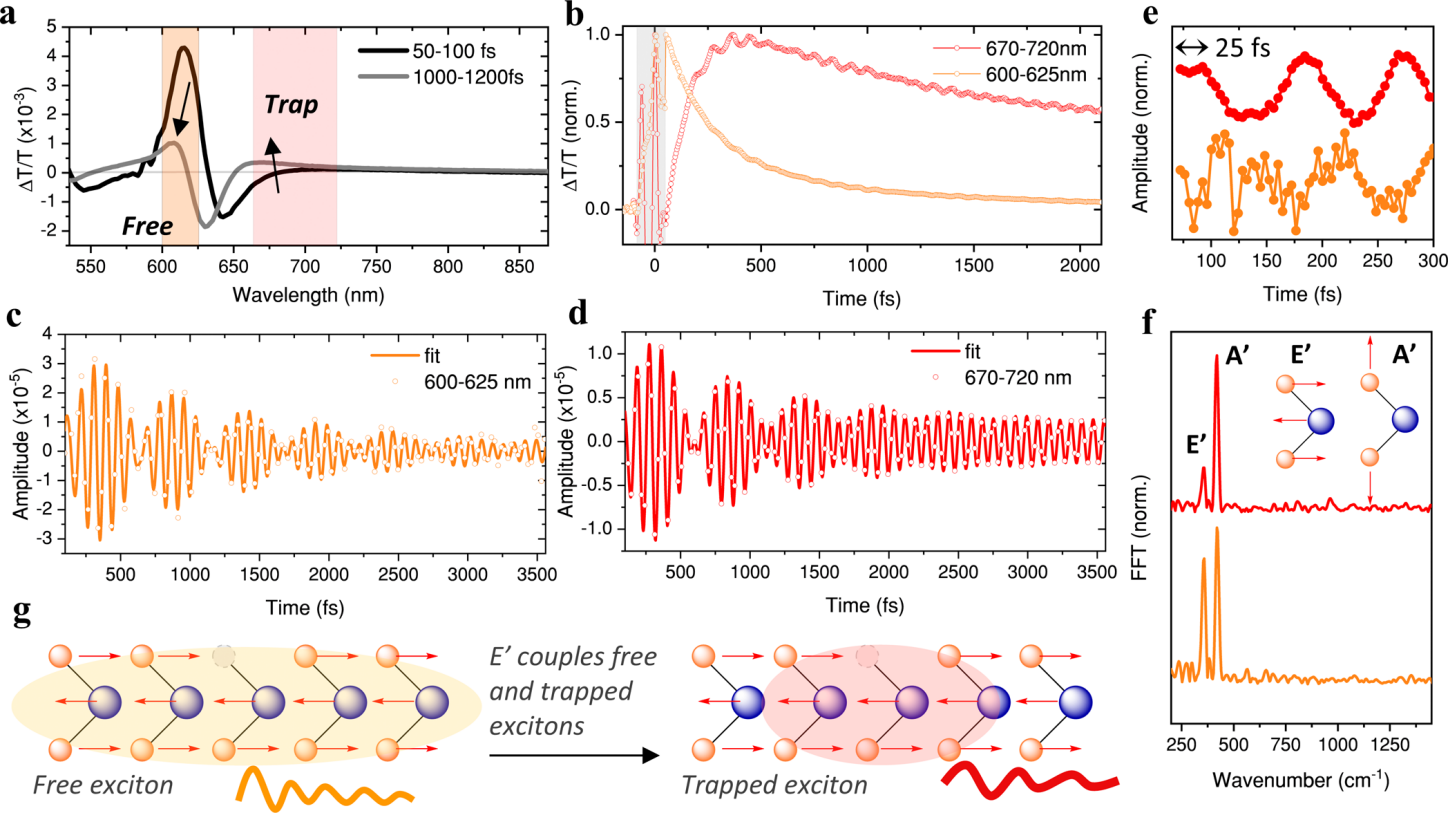
Exciton trapping modulates phonon coherences. (a) Transient absorption spectra of *n*-BuLi treated WS_2_ monolayer. (b) Kinetics integrated between 600–625 nm (free exciton in orange) and 670–720 nm (trapped exciton in red). Gray transparent box indicates coherent artifact. (c) Electronic-decay free oscillation and its corresponding damped sinusoid function fit for the free exciton. (d) Electronic-decay free oscillation and its corresponding damped sinusoid function fit for the trapped exciton. (e) Early time scale electronic-decay free oscillations. (f) Fast Fourier transform (FFT) over the complete time range. (g) Schematic 1D illustration of the coupling/promoting role of the E’ mode to drive exciton trapping.

Figure 3b shows the normalized kinetic traces of both free (orange) and trapped (red) excitons. As the free exciton bleach decreases in intensity, the trapped exciton signal increases. The latter is fitted to a biexponential function with time constants of τ_1_ = 114 fs and τ_2_ = 1.3 ps, that represent the trapping and recombination time scales, respectively. The trapping lifetime is slightly shorter than previously reported for untreated WS_2_ (∼300–400 fs),^46,48^ but notably shorter than that observed in MoS_2_ (1–2 ps).^22,35^ The concomitant reduction in the free exciton A signal and increase in trapped exciton signal supports the assignment of the emerging spectral features to excitons trapped at defect sites.

The kinetics in Figure 3b reveal oscillations superimposed on the electronic dynamics. The electronic dynamics are removed by the biexponential fit to acquire the pure oscillatory traces for the free and trapped excitons in Figure 3c,3d, respectively. These ∼80 fs period oscillations represent phonon coherences generated by the ultrafast pump (10 fs), which modulate the transient absorption spectrum in time. Strikingly, the conservation of phonon coherence during the exciton trapping process is inconsistent with an incoherent, hopping-like mechanism, which would be expected to destroy any phonon coherence.^49–52^ A closer inspection of the first 300 fs reveals a ∼25 fs delay in the buildup of coherence associated with trapping (Figure 3e), suggesting a trapping-mediated transfer of coherence. The fast Fourier transform (FFT) of these oscillations reveal both the Raman-active E’ (356 cm^–1^) and A’ (420 cm^–1^) modes (Figure 3f), and are close to the ground-state Raman mode frequencies (Figure 1c). The assignment of the A’ mode is further discussed in Supporting Note 1 (Figures S2–S4).

We now analyze the phonon coherences in more detail to elucidate the physical interpretation of the conserved coherence. We fitted the electronic-decay free oscillatory traces to a sum of two exponentially damped sinusoid functions (eq 1)

$$f\left(t\right)=A_{1}e^{-t/\tau_{1}}\sin\left(2\pi \times \omega_{1}t-\phi_{1}\right)+A_{2}e^{-t/\tau_{2}}\sin\left(2\pi \times \omega_{2}t-\phi_{2}\right)$$ 1

Where *A*_1_ and *A*_2_ represent the amplitudes, ω_1_ and ω_2_ the frequencies, τ_1_ and τ_2_ the dephasing times (often loosely referred to as “lifetimes”), and ϕ_1_ and ϕ_2_ the phases, of the A’ and E’ oscillations, respectively. The fitted parameters are provided in Table 1 and the residuals in Figure S5. Traditionally, phonon dephasing has contributions from defect-phonon scattering induced by the local potential (τ_defect_), phonon–phonon scattering through anharmonicity (τ_anharmonic_) and phonon-carrier scattering (τ_carrier_),^24^ resulting in a total dephasing time (τ_total_)^−1^ = (τ_defect_)^−1^ + (τ_anharmonic_)^−1^ + (τ_carrier_)^−1^. The fitting provides two key observations: (1) the E’ mode dephases faster (980 fs for free exciton) compared to the A’ mode (2.4 ps) (2) Trapping induces a faster dephasing of E’ (741 fs). Our sliding window FFT analysis (Figure S6) supports both observations. The fittings were repeated for more spectral ranges with their parameters provided in Figure S7.

**Table 1:** Damped Sinusoid Fitting Results^a^ [Disp-formula eq1]

mode i (spectrum)	ν_i_ (cm^–1^)	A_i_ (× 10^–5^)	τ_i_ (fs)	ϕ_i_ (rad)
A’ (free)	420 ± 0.3	1.3 ± 0.08	^b^2386 ± 392	4.4 ± 0.06
A’ (trap)	419 ± 0.1	0.5 ± 0.01	^b^4770 ± 454	1.6 ± 0.03
E’ (free)	356 ± 0.3	2.8 ± 0.12	980 ± 57	6.1 ± 0.04
E’ (trap)	356 ± 0.3	1.0 ± 0.03	741 ± 30	4.0 ± 0.03

a The electronic-decay free oscillatory traces in Figure 3c,d are fitted to equation 1.

b Due to the limited temporal measurement range, this value should be interpreted with caution.

The faster dephasing of the E’ mode compared to the A’ may arise from their distinct symmetries. As an in-plane vibrational mode, E’ may be expected to couple more strongly to local defects than the out-of-plane A’ mode, leading to more rapid dephasing.^53^ This is analogous to the faster dephasing of C–H stretching compared to C–H bending in a diamond nitrogen-vacancy center.^27^ However, optical phonon lifetimes in TMD monolayers are typically on the order of a few picoseconds^54–56^ and are primarily governed by optical-acoustic phonon scattering.^57,58^ In this context, the E’ mode may exhibit shorter lifetimes due to its more efficient phonon–phonon Umklapp scattering enabled by its greater sensitivity to in-plane strain compared to the A’ mode.^59^ Depending on excitation conditions, phonon-carrier^60–62^ scattering may also contribute to the phonon dephasing. For example, phonon-carrier induced fast dephasing could explain the absence of the E’ mode in the on-resonant FFT spectra of MoS_2_ ^54^ and WSe_2_.^55^ For MoS_2_, the E’ mode lifetime increases to 7 ps under off-resonant conditions.^63^ Anisotropic electronic distributions and their corresponding momentum relaxation processes correlate with short lifetimes for nonsymmetric modes.^60^

Whereas phonon–phonon (anharmonic) and phonon-defect contributions may in principle be decoupled with temperature and defect density dependence,^24^ respectively, broadband IVS enables spectral selectivity of phonon-carrier scattering. As such, we can conclude that the exciton trapping induces a faster E’ dephasing (Table 1), either (i) due to an increased exciton–phonon coupling due to the smaller size of the trapped exciton or (ii) because the nonadiabatic transfer associated with trapping causes additional dephasing (Figure 3g). In fact, coupling/promoter modes that drive nonadiabatic transfer experience more rapid dephasing at surface crossings.^51,64–68^ Although the A’ mode appears to be longer-lived as a result of trapping, the exact lifetime values of the A’ mode are not accurate due to the limited temporal range of the measurement. The spectral insensitivity of the A’ and E’ Raman frequencies indicates that the vibrational manifold is only weakly perturbed by the trapping, implying a similar size of the trapped exciton compared to its free size (∼1–2 nm),^69^ consistent with explanation (ii). Indeed, the trapping potential has to be sufficiently broad to capture excitons efficiently.^17^ Consistently, we do not observe any local defect-related modes, in contrast to the IVS results on MoSe_2_ from Bae et al.^70^

Electron and hole trapping at defect sites has been traditionally described by a multiphonon emission process involving “promoting” (or coupling) and “accepting” modes that couple the delocalized charge carrier to the defect state and accept the excess energy released by this process, respectively (Figure 4).^14,71,72^ This mechanism allows nonradiative transitions between states with energy differences much larger than a single available phonon energy to occur. In the present case, the 0.15 eV gap between trapped and free exciton states exceeds any optical phonon mode in WS_2_. Note that a single mode can act as both a promoting and accepting mode. The first step typically involves an activation barrier and is therefore temperature dependent, making the overall process incoherent. Such a classical picture is not consistent with our observations of ultrafast exciton trapping accompanied by the conservation of phonon coherence.

**Figure 4:**
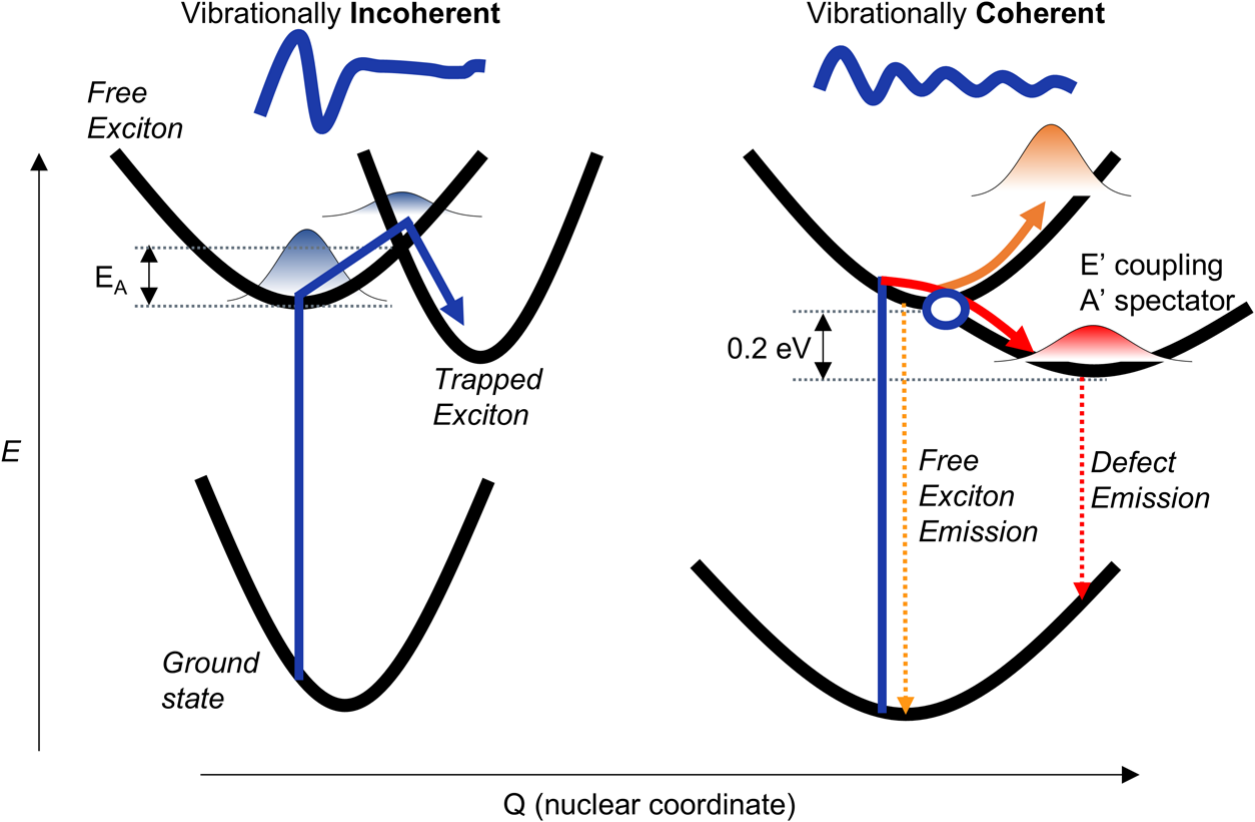
Configuration coordinate diagram for ultrafast coherent exciton trapping. In the case of traditional thermally activated trapping, an activation barrier (E_A_) precedes the surface crossing leading to an incoherent process. For a barrierless trapping the coherence generated in the free excitonic state may be transferred to the trapped excitonic state. Coupling modes experience stronger dephasing than tuning or spectator modes as they nonadiabatically couple the free and trapped excitonic states via a conical intersection (indicated with a blue circle). Conventionally, modes that couple trapped to free states are classified as promoter modes, whereas modes absorbing the excess energy as a result of trapping are called accepting modes.

Instead, we draw inspiration from a mechanistic feature common in molecular photochemistry, a conical intersection, to explain our observations. These are crossings between adiabatic electronic states in nuclear configuration space that enable coherent transfer of vibronic wavepackets.^73–80^ Such nonadiabatic transitions inherently involve a breakdown of the Born–Oppenheimer approximation, in stark contrast to the incoherent, thermally activated multi-phonon emission mechanism. While vibronically coherent processes are well-known in molecular systems—such as singlet fission^81^ and rhodopsin photoisomerization^82^—they remain largely unexplored in the context of defect trapping in solids. The persistence of vibronic coherence during exciton localization in monolayer WS_2_ points to a conical intersection-mediated mechanism involving vibronic transfer across the free and trapped excitonic states.

Consistent with vibronic transfer of coherence, there is a build-up time of the trapped exciton phonon coherence, but not in the free exciton phonon coherence (Figure 3e).^83^ This delay is associated with a phase shift with respect to the free exciton coherence (Δϕ = 0.9 ± 0.3π for A’, Δϕ = 0.7 ± 0.2π for E’ at 100 fs, see Table 1),^83^ which also confirms the excited-state nature of the trapped exciton coherence. We further rule out ground-state coherence by the resonance conditions (Supporting Note 1) and the ∼π phase flip across the spectrum (Figures S7 and S8).^30,84^ Although reaction-driven coherence could be associated with a spectral π phase flip as well, we exclude any reaction-driven generation of the coherences^65,66,84,85^ in the trapped exciton based on the slower trapping time scale (114 fs) compared to the periods of E’ (95 fs) and A’ (79 fs) modes. Furthermore, although the trapped and free exciton signals are spectrally convoluted, the node appears to be located in-between these, instead of the maximum of the trapped exciton. Finally, we disregard an adiabatic vibrationally coherent relaxation mechanism, such as self-trapped exciton formation, to explain our observations. Here, slow dephasing is dominated by phonon–phonon and phonon-defect interactions.^85–88^ However, sulfur vacancies introduce midgap states, which can be excited directly (Figure S4), and lead to an additional bright emitting state rather than a single broadened and stokes-shifted emission typically associated with self-trapping.^89,90^

Analogously to the promoting and accepting classification of relevant modes in carrier trapping, conical intersections are characterized by coupling and tuning modes, although with different meanings. The coupling and tuning modes are both promoting in character as they mediate nonadiabatic coupling and the energy gap between the two states, respectively. Coupling modes,^51,64–67^ and sometimes in more general vibronic modes,^68^ have rather rapid dephasing times due to strong anharmonicities present at the conical intersection,^91^ whereas tuning modes or spectator modes (modes without any functional role) are longer-lived.^92,93^ The E’ mode has the correct symmetry to mediate the in-plane real space shrinking of the free to trapped exciton wave function (Figure 3g) (without any change in wavevector *k* due to the nondispersive nature of the defect bands^38^) and could therefore have coupling character (Supporting Note 2). The A’ mode, on the other hand, vibrates out-of-plane and thus can not couple the two excitonic states (although it does modulate the free excitonic transitions via amplitude modulation as discussed below). These assignments are consistent with their different dephasing behavior. Whereas the large lifetime and the lifetime extension associated with trapping for the A’ mode (Table 1) indicates a spectator role, the trapping-enhanced dephasing of E’ is consistent with its coupling character.

The distinct relative phases between E’ and A’ in the trapped exciton population compared to the free exciton population (Δϕ = 0.76π and 0.54π, respectively at 100 fs) suggest that the transfer of coherence associated with exciton trapping happens at different rates for both modes, confirming their different roles (Table 1 and Figure S7). On the contrary, the phase for both E’ and A’ remains constant across the exciton A absorption spectral region. This is manifested as an absence of a node at the exciton A maximum (Figure S1), implying *amplitude* modulation^94^ of the excitonic transitions via non-Condon effects rather than *frequency* modulation,^95^ which has also been observed for exciton C in MoS_2_.^54^ By extrapolating the fitted oscillatory traces to *t* = 0 fs, we can conclude that the A’ mode is generated in the displacive limit or via resonant impulsive stimulated Raman scattering (rISRS) as it follows a cosine function (Figure S9).^96^ The E’ mode is neither a pure impulsive mode, nor a pure displacive mode, based on its ‘intermediate phase’. Such an intermediate initial phase indicates a failure of the extrapolation, which assumes no significant changes in electronic population in the first 100 fs and is consistent with its strong coupling to the exciton trapping process. A discussion of unifying Raman theories that can explain these ‘intermediate phases’ ^97–99^ is beyond the scope of this article.

To further substantiate the proposed mechanism of vibronically coherent exciton trapping via a defect-induced conical intersection, future theoretical work should aim to explicitly identify such structures in the presence of point defects in TMD monolayers. While defect-induced conical intersections have been studied in medium-sized nanoclusters,^100,101^ their role in extended crystalline systems remains largely unexplored. In nanoclusters, defect-induced conical intersections may drive fast nonradiative decay of trapped excitons to the ground state.^100,101^ In contrast, trapped excitons in WS_2_ decay radiatively with long lifetimes^34,36^ making such a decay channel unlikely in our case. Finally, we note that the presence of free exciton emission despite the ultrafast trapping time scale is likely a result of the spatial inhomogeneity of the defect distribution, requiring slow carrier diffusion which is outcompeted by emission.

## Conclusions

In this study, we have used IVS to investigate exciton trapping in chemically treated WS_2_ monolayers grown by MOCVD. We find that the phonon coherences of the A’ and E’ Raman modes survive the (∼100 fs) trapping process, demonstrating that this process is vibrationally coherent, in contrast to the classical picture of incoherent carrier trapping. This vibrationally coherent process suggests a conical intersection may connect the free and trapped excitonic states, mirroring fast and efficient excitonic processes seen in molecular systems, such as the photoisomerization of rhodopsin which underlies vision. The faster dephasing of the E’ mode when coupled to the trapped exciton compared to the free exciton suggests a promoting/coupling role of the trapping process through the conical intersection. Future work should explore how such vibronic pathways depend on layer thickness, type of defect, momentum-space valleys and spatial distribution, and whether they represent a general mechanism for carrier trapping across other low-dimensional materials, bulk semiconductors and nanomaterials—particularly in cases where the trapping occurs on ultrafast time scales.^22,35,102–107^ This represents a previously unexplored role for optical phonons in TMDs, which through momentum conservation control moiré interlayer exciton formation,^108^ exciton dissociation^109^ and emission of indirect excitons.^110^ Trapping has been known to induce certain nuclear rearrangements,^105^ local defect mode activation^70^ and a piezoresponse by triggering acoustic modes,^111^ but the dynamical mechanisms of exciton trapping have been rather unexplored experimentally. Point defect vibronic coherences are potential carriers of quantum information and could be exploited for single photon emitters and related light-defect coupled applications.

## Methods

### Materials

#### MOCVD growth of WS_2_

A 4-in. diameter horizontal hot-wall MOCVD system with three heating zones was used for the synthesis of WS_2_ films on sapphire substrates. Tungsten hexacarbonyl (THC, purity 99.99%, Sigma-Aldrich, 472956) and diethyl sulfide (DES, purity 98%, Sigma-Aldrich, 107247) served as the tungsten and sulfur sources, respectively. A mixture was prepared by dissolving 0.7 g of THC in 100 mL of DES and was stored in a stainless-steel bubbler. An additional supply of DES was kept in a separate bubbler. Both bubblers were maintained at a constant pressure of 800 Torr and room temperature. A Si wafer with 100 nm-thick oxide was horizontally placed in a quartz plate and loaded into the first zone of the furnace after the cleaning process using acetone and isopropanol. Before growth, the system was ramped up to 600 °C for 1 h in a mixture of 1600 sccm of Ar and 20 sccm of H_2_ at a total pressure of 21.3 Torr. During the growth phase, the flow rate of the carrier gas was reduced to 720 sccm of Ar and 5 sccm of H_2_, maintaining a pressure of 11.3 Torr. A precise amount of 2 sccm of the THC/DES mixture and 3 sccm of DES were injected into the chamber. After 10 h of growth, the injection of the mixed precursor was halted, and the system was slowly cooled down under 1600 sccm of Ar and 0.5 sccm of H_2_ while the flow of DES was kept at 0.3 sccm to minimize the generation of sulfur vacancies. When the system reached 300 °C, the DES line was closed, and the WS_2_ film remained in the Ar/H_2_ environment until the system cooled down to room temperature.

#### *n*-Butyllithium Treatment

WS_2_ flakes and films were immersed in *n*-butyllithium (1.6 M, Sigma-Aldrich) for 3 h, rinsed with anhydrous hexane (Sigma-Aldrich), and dried. This process was conducted in an N_2_ glovebox. The treated samples were subsequently washed with deionized water (DI), acetone, and isopropyl alcohol (IPA).

#### Mechanical Exfoliation

Mechanical exfoliation was performed using a PDMS-assisted method. WS_2_ flakes (2D semiconductor) were exfoliated onto PDMS (Gel-Pak) using blue tape (Ultron Systems, Inc.), and monolayer flakes were identified under an optical microscope. The selected monolayer was then transferred onto ultrathin borosilicate glass.

### Spectroscopy

#### Photoluminescence and Raman Microscopy

The steady-state Raman measurements were conducted using a Renishaw inVia Raman confocal microscope with a 532 nm excitation laser under ambient conditions. Emission was collected using a 20× long-working-distance objective lens in streamline mode and dispersed by an 1800 l/mm grating with 0.5% of the laser power (<2 μW).

#### Impulsive Vibrational Spectroscopy

The IVS experiments were performed at room temperature using a custom-built pump–probe setup with a temporal resolution of 10 fs. A broadband white light continuum (WLC) spanning 530–950 nm was generated by pumping a YAG crystal with a 1030 nm fundamental wavelength from a Yb:KGW amplifier laser (Light Conversion Pharos, 14.5 W, 38 kHz repetition rate, 200 fs). This WLC served as the probe beam and was used as a seed to make the pump pulses. The pump beam was produced through noncollinear optical parametric amplification (NOPA) of the 1030-WLC using either the second (515 nm) or third harmonic (343 nm), which were generated by an automatic harmonic generator (Light Conversion HIRO). These pulses achieved durations of 10 fs with spectra spanning 500–650 nm when amplified by the third harmonic, and 11 fs with spectra spanning 650–900 nm when amplified by the second harmonic. Compression of the pump pulses was achieved using chirped mirrors and wedge prisms (Layertec). The spatiotemporal profiles of these pulses were characterized via second-harmonic generation frequency-resolved optical gating (SHG-FROG). Differential transmission spectra were obtained by modulating the pump beam at 9 kHz with a chopper wheel. A computer-controlled piezoelectric translation stage (PhysikInstrumente) with a 4 fs step size was used to set the pump–probe delay. To minimize pump scatter, an ultrabroadband wire grid polarizer (Thorlabs) was placed in the detection path. The transmitted probe was measured using a grating spectrometer equipped with a Silicon camera (Entwicklungsbüro Stresing) operating at 38 kHz and a 550 nm blazed grating. The incident pump fluence was ∼ 10 μJ/cm^2^, corresponding to an estimated initial carrier density of *n*_0_ ∼ 1.4 × 10^12^ cm^–2^, below the Mott density of *n*_Mott_ ∼ 2–3 × 10^12^ cm^–2^.^112^

Before the time-domain analysis, background removal and chirp correction were applied to the kinetic traces. Then, biexponential fitting was applied to acquire the electronic-decay free oscillatory traces that represent the phonon coherences. To remove any coherent artifact contributions, these fits were carried out from 70 fs. The resulting traces were either fitted to eq 1 to gain further time-domain information or a FFT analysis was carried out to determine the Raman frequencies. For the first, a 0.01 Hz high-pass fast Fourier transform (FFT) filter was first applied to eliminate artificially low frequencies due to background noise. The FFT analysis was carried out following zero-padding and the application of a Blackman window to reduce ringing.

## Supplementary Material



## Data Availability

The data that support the findings of this study is available to download at the University of Cambridge’s Apollo Repository [DOI to be added at acceptance].

## Notes

Photoluminescence and Raman spectroscopy of mechanically exfoliated WS_2_, additional analysis and discussion of impulsive vibrational spectroscopy data (PDF)
