# Supplementary material for: Vibronically Coherent Exciton Trapping in Monolayer WS2
Source: ACS Nano. 2025 Jul 21;19(29):26942–52. doi: 10.1021/acsnano.5c08533 (PMC12312154; doi:10.1021/acsnano.5c08533)
Supplement: Supplementary file 1 [file nn5c08533_si_001.pdf]

Supporting Information

Vibronically coherent exciton trapping in monolayer WS_2

Authors: Yorrick Boeije[1,2], Anh Tuan Hoang[3], Juhwan Lim[2,4], Samuel D. Stranks[1,2], Manish Chhowalla[4], Eric Pop[3,5,6], Andrew J Mannix[3], Akshay Rao[2]*

Affiliations:

[1]Department of Chemical Engineering and Biotechnology, University of Cambridge; Cambridge, UK.

[2]Department of Physics, Cavendish Laboratory, University of Cambridge; Cambridge, UK.

[3]Department of Materials Science & Engineering, Stanford University, Stanford, California 94305, United States.

[4]Department of Materials Science & Metallurgy, University of Cambridge; Cambridge, UK.

[5]Department of Electrical Engineering, Stanford University, Stanford, California 94305, United States.

[6]Department of Applied Physics, Stanford University, Stanford, California 94305, United States.

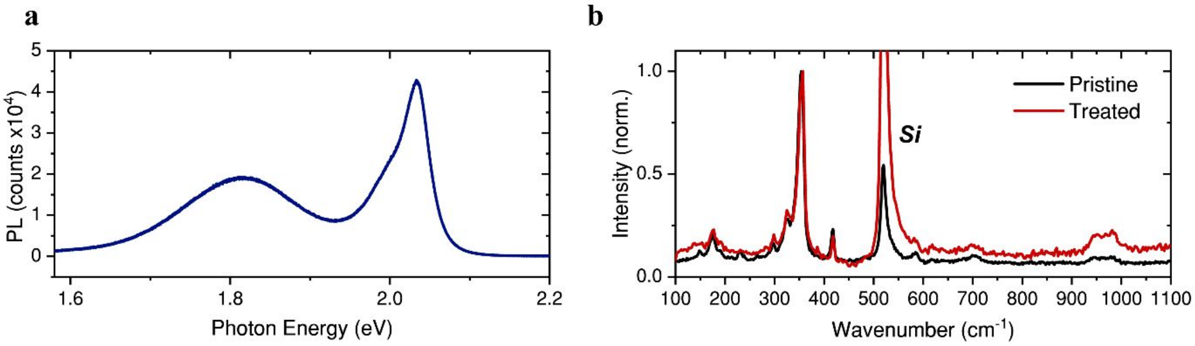

Supplementary Figure S1. (a) Room temperature photoluminescence spectrum (λ_{exc} = 532 nm) of a *n*Bu-Li treated monolayer WS_2 prepared with mechanical exfoliation. (b) Raman spectra of treated and untreated films. The silicon peak is indicated.

Supplementary Note 1

We assign the 419 cm^{-1} mode to A', despite its proximity to a sapphire mode, based on the clear nodal pattern in the Raman map, emphasizing its coupling to excitonic transitions (as is also observed for E') (Fig. S2). In contrast, the sapphire mode is insensitive to probe wavelength as it is probed off-resonantly (Fig. S3).[1] Furthermore, other sapphire modes (576 cm^{-1} and 750 cm^{-1}) do not appear in the WS_2 fast Fourier transform (FFT) spectra. Upon edge excitation of the WS_2 monolayer (Fig. S4), the disappearance of the 419 cm^{-1} nodal pattern, the absence of any E' mode and appearance of the other sapphire modes suggest that the off-resonant impulsive stimulated Raman scattering (ISRS) mechanism dominates for this excitation condition. In contrast, the appearance of A' and E' modes for on-resonant excitation of WS_2 (Fig. S2) suggests that these modes are generated via resonant-ISRS or via the displacive excitation of coherent phonons (DECP) mechanism.[2,3] The spectral π-phase flip of both A' and E' modes present when WS_2 is excited on-resonantly, supports their excited state nature, as ground state wavepackets generated by the off-resonant ISRS mechanism have no π-phase flip (Fig. S8).[4]

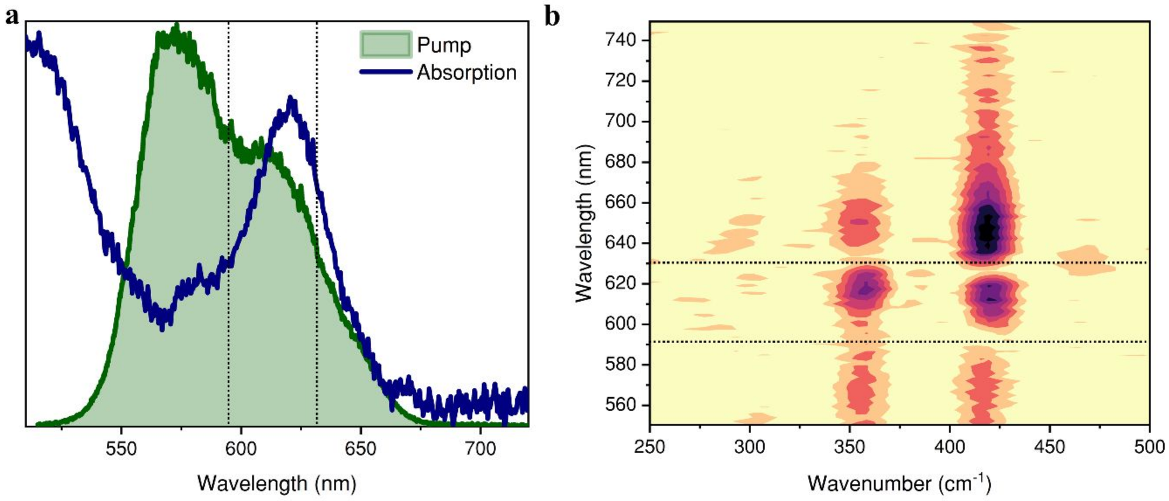

Figure S2. (a) Green broadband pump and absorption spectrum. (b) Room temperature ultrafast Raman map of *n*Bu-Li treated WS_2 film. The two nodes are marked with a dotted black line, and also indicated in the absorption spectrum.

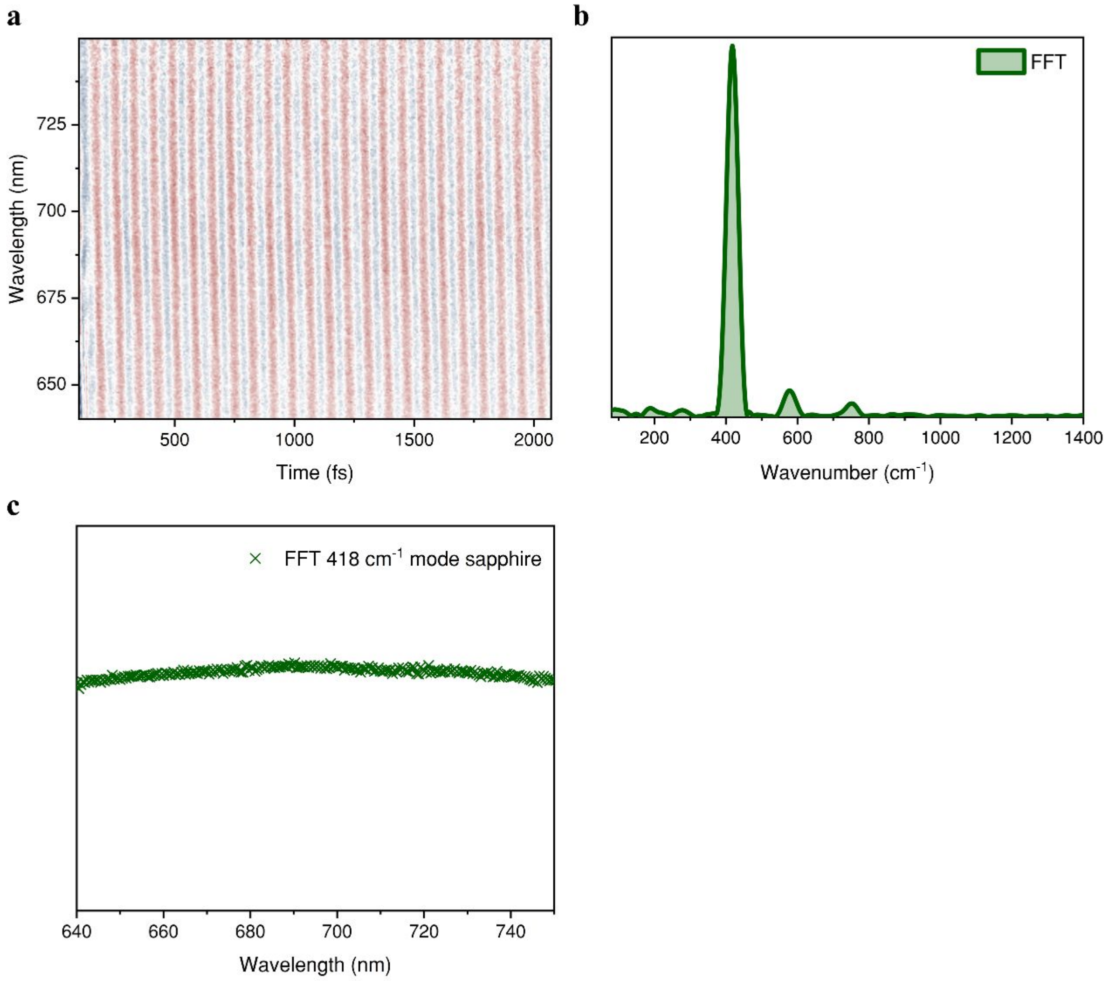

Figure S3. (a) Transient absorption map of sapphire substrate pumped with same broadband pulse as shown in Fig. S2a. (b) The FFT spectrum of the 670-720 nm probe region. (c) FFT intensity dependence on probe wavelength of 418 cm^{-1} mode.

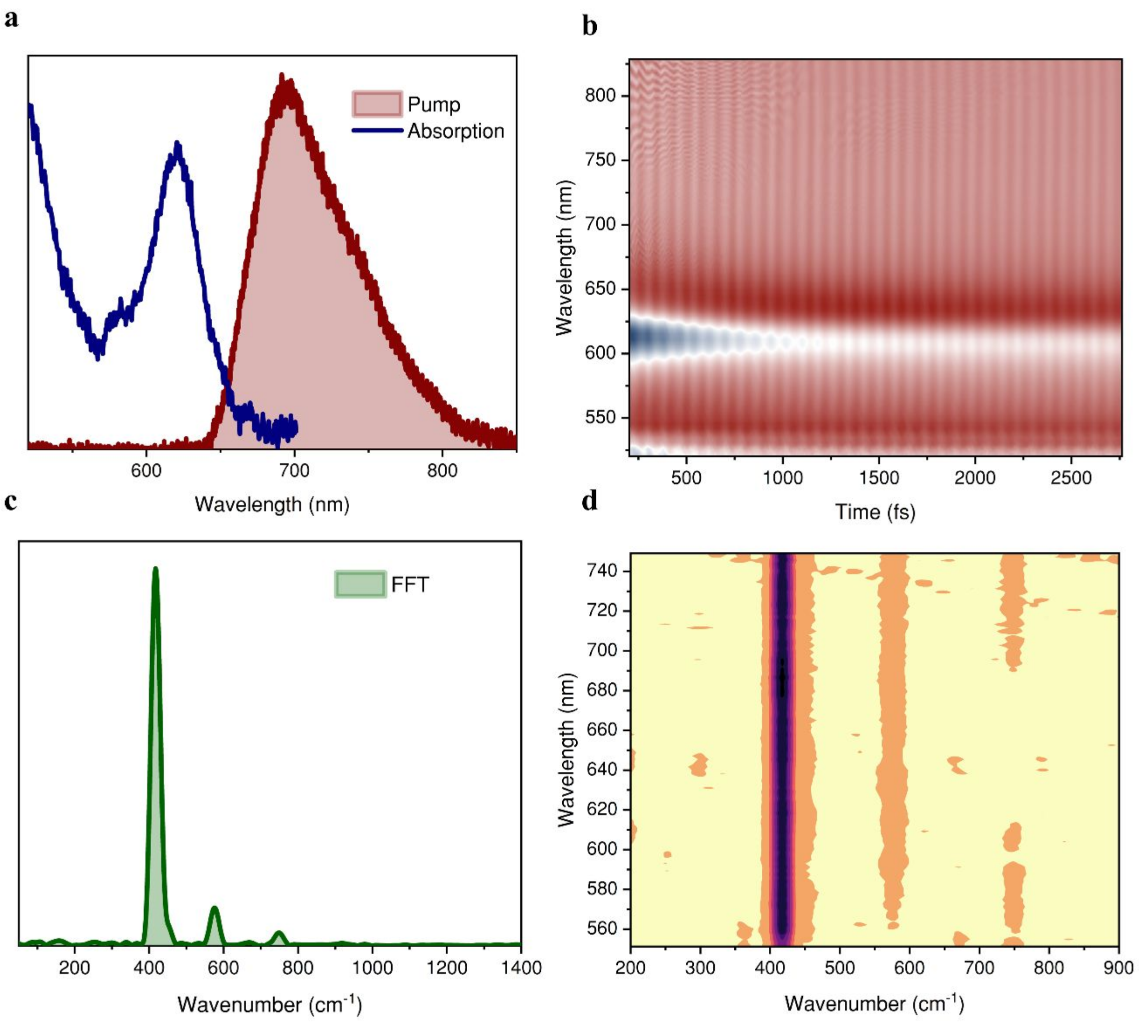

Figure S4. (a) Red broadband pump and absorption spectrum. (b) Transient absorption map of *n*Bu-Li treated WS_2 film. (c) The FFT spectrum of the 670-720 nm spectral region. (d) FFT map.

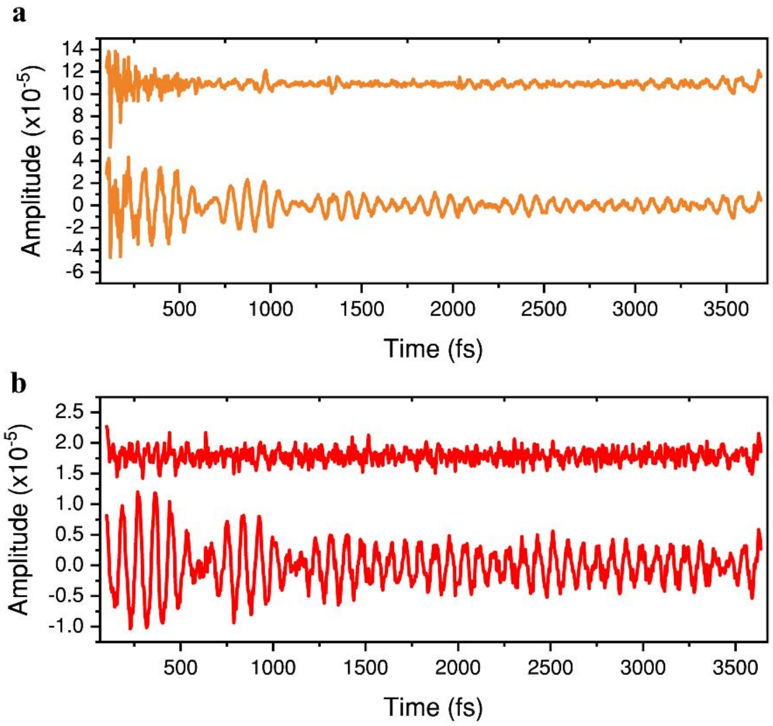

Figure S5. (a) Residuals overlaid on the electronic decay-free transient of the free exciton. (b) Residuals overlaid on the electronic decay-free transient of the trapped exciton. (The original transients are also shown in Fig. 3c and 3d of the main text.)

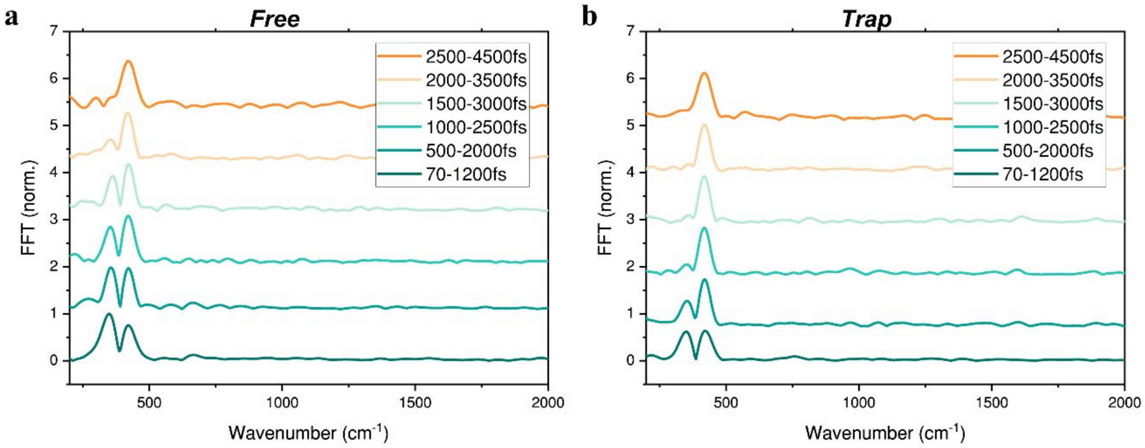

Figure S6. (a) Sliding-window FFT for free exciton. (b) Sliding-window FFT for trapped exciton.

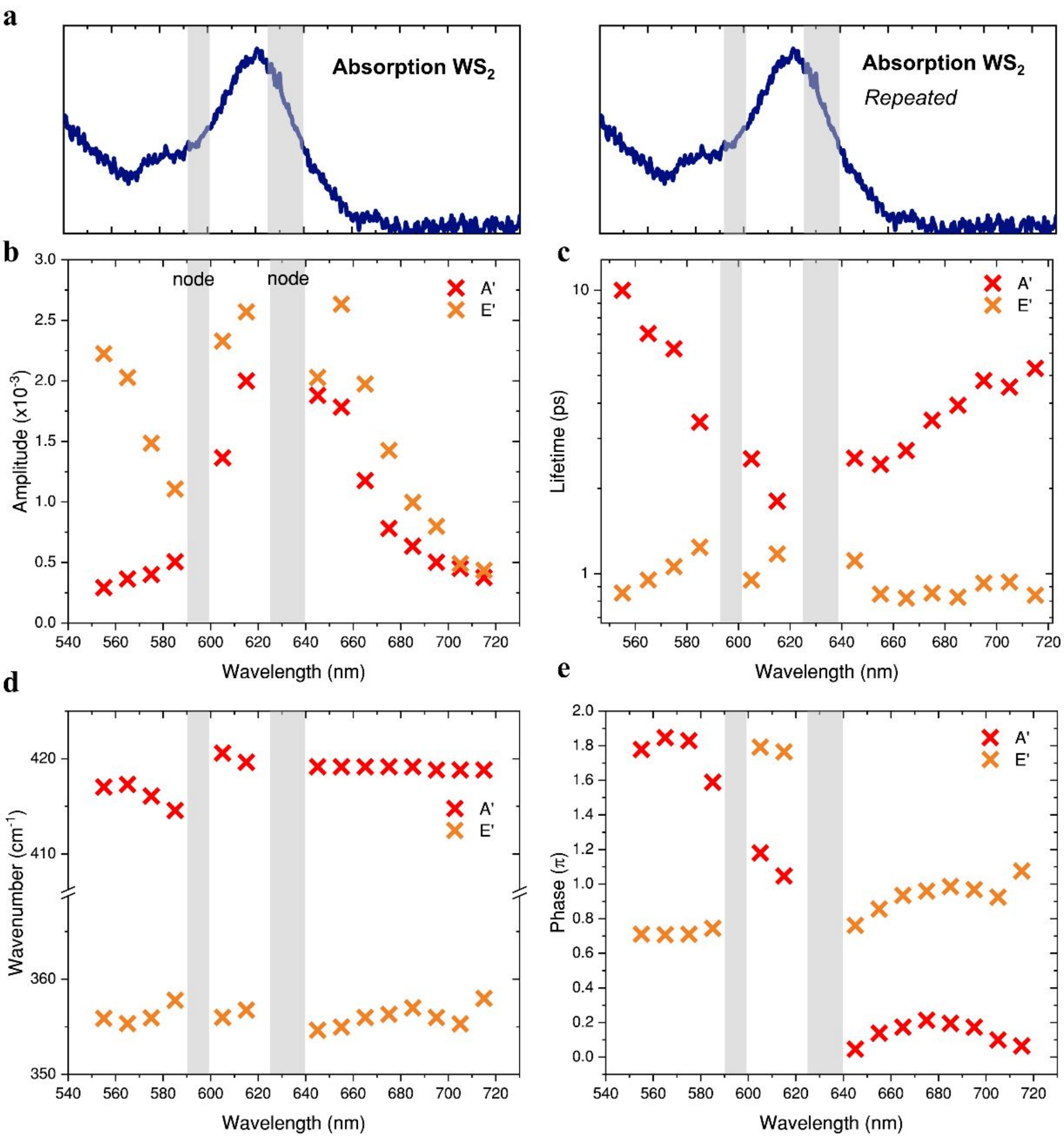

Figure S7. Spectral-dependence of damped sinusoid fitting. (a) Nodal spectral regions are indicated with grey boxes and are aligned with the absorption spectrum of WS_2. (b) Spectral dependence of amplitude. (c) Spectral dependence of lifetime. (d) Spectral dependence of wavenumber. (e) Spectral dependence of phase. Representative oscillatory traces are provided in Fig. S5.

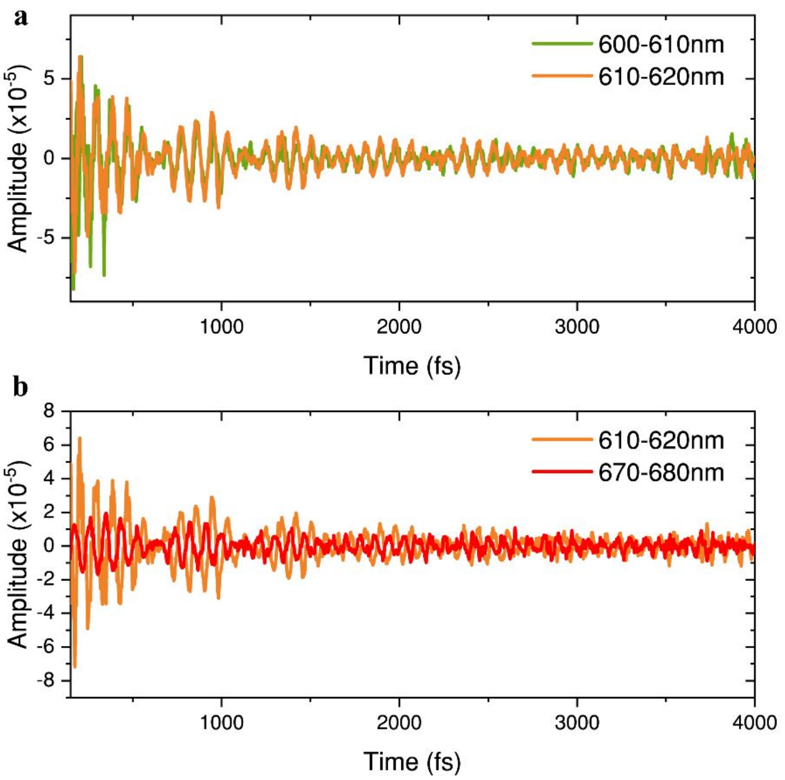

Figure S8. (a) In-phase oscillatory traces for two spectral slices of exciton A. (b) Oscillatory traces between exciton A and trapped exciton show out-of-phase relationship.

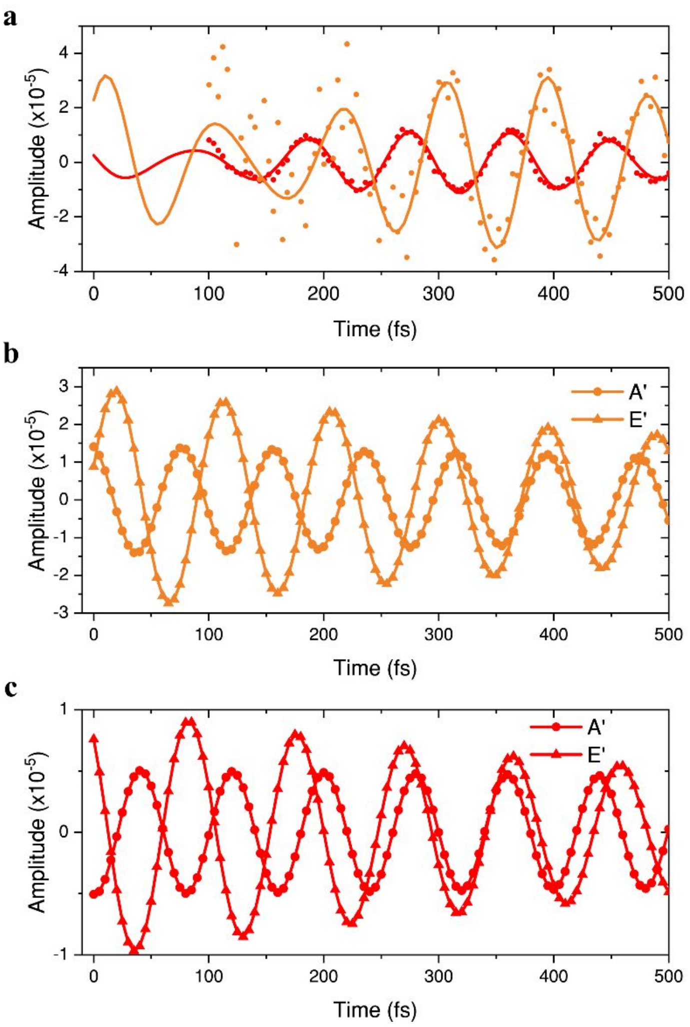

Figure S9. (a) Oscillatory traces of free (600-625 nm, orange) and trapped (670-720 nm, red) excitons (Fig. 3c and 3d in the main text) and their extrapolated damped sinusoid fits to $t = 0$ fs. Fitted parameters are provided in Table 1 of the main text. (b) Mode-specific extrapolated fits for free exciton. (c) Mode-specific extrapolated fits for trapped exciton.

Supplementary Note 2

Ignoring defect-induced symmetry breaking, a group theory based argument can be made using the C_{3h} point group at the K band edge.[5] As the valence band maximum and conduction band minimum at the K point have E’ and A’ symmetry, respectively, the in-plane exciton has E’ symmetry. Assuming the trapped exciton retains the same symmetry, the non-adiabatic coupling matrix element $\left\langle \varphi_{trapped} \middle| \frac{\partial}{\partial Q} \middle| \varphi_{free} \right\rangle$ is non-zero as the direct product E’ □ E’ □ E’ contains the totally symmetric irreducible representation of C_{3h}, A_1’.

References

(1) Liebel, M.; Schnedermann, C.; Wende, T.; Kukura, P. Principles and Applications of Broadband Impulsive Vibrational Spectroscopy. *J. Phys. Chem. A* **2015**, *119* (36), 9506–9517. DOI:10.1021/ACS.JPCA.5B05948.

(2) Zeiger, H. J.; Vidal, J.; Cheng, T. K.; Ippen, E. P.; Dresselhaus, G.; Dresselhaus, M. S. Theory for Displacive Excitation of Coherent Phonons. *Phys. Rev. B* **1992**, *45* (2), 768–778. DOI:10.1103/PhysRevB.45.768.

(3) Sayers, C. J.; Genco, A.; Trovatello, C.; Conte, S. D.; Khaustov, V. O.; Cervantes-Villanueva, J.; Sangalli, D.; Molina-Sanchez, A.; Coletti, C.; Gadermaier, C.; Cerullo, G. Strong Coupling of Coherent Phonons to Excitons in Semiconducting Monolayer MoTe2. *Nano Lett.* **2023**, *23* (20), 9235–9242. DOI:10.1021/ACS.NANOLETT.3C01936.

(4) Kumar, A. T. N.; Rosca, F.; Widom, A.; Champion, P. M. Investigations of Amplitude and Phase Excitation Profiles in Femtosecond Coherence Spectroscopy. *J. Chem. Phys.* **2001**, *114* (2), 701–724. DOI:10.1063/1.1329640.

(5) Xiao, D.; Liu, G. Bin; Feng, W.; Xu, X.; Yao, W. Coupled Spin and Valley Physics in Monolayers of MoS 2 and Other Group-VI Dichalcogenides. *Phys. Rev. Lett.* **2012**, *108* (19). DOI:10.1103/PHYSREVLETT.108.196802.
